# Fabrication of cubic PtCu nanocages and their enhanced electrocatalytic activity towards hydrogen peroxide

**DOI:** 10.1186/1556-276X-9-68

**Published:** 2014-02-10

**Authors:** Liangliang Tian, Xiaohui Zhong, Wanping Hu, Bitao Liu, Yunfeng Li

**Affiliations:** 1 Department of Research Center for Materials Interdisciplinary Science, Chongqing University of Arts and Sciences, Chongqing 402160, China; 2 Department of Chemical Engineering, University of Missouri, Columbia, MO, USA

**Keywords:** Hydrogen peroxide, PtCu nanocage, Biosensor

## Abstract

Cubic PtCu nanocages (NCs) were successfully synthesized through a redox reaction using cuprous oxide (Cu_2_O) as a sacrificial template and reducing agent. The porous PtCu NCs were composed of amounts of PtCu nanograins with an average particle size of 2.9 nm. The electrocatalytic performance of the PtCu NC electrode towards H_2_O_2_ was studied by cyclic voltammetry (CV) and chronoamperometry. The prepared PtCu NC electrode exhibited excellent electrocatalytic activity towards H_2_O_2_, with a wide liner range from 5 μM to 22.25 mM, a relatively high sensitivity of 295.3 μA mM^-1^ cm^-2^, and a low detection limit of 5 μM (S/N = 3). The hollow porous nanostructure has potential applications in biosensors.

## Background

Quantitatively accurate and fast determination of H_2_O_2_ is extremely important in the field of food industry, pharmaceutical, clinical, industrial, and environmental analyses [1]. Among the several analytical techniques, electrochemical analysis is widely used due to the fast response, high sensitivity, and excellent selectivity [2,3]. Although enzyme-modified electrode is always used to build H_2_O_2_ biosensor due to its high selectivity, the enzymatic biosensors still suffer from the insufficient stability which originated from the nature of enzymes [4]. Therefore, the study of nonenzymatic H_2_O_2_ sensors is aroused in this field. It is known that platinum shows excellent electroactivity because of the efficient electron transfer rate [5,6]. Platinum with special morphologies, such as spherical [7], cubic [8], nanowires [9], nanoflowers [10], have been reported to construct biosensors. In addition, specific surface area is also a key factor affecting the performance of the biosensor. Therefore, how to increase the specific surface area is the focus in scientific research. Hollow structures have attracted extensive attentions for their special frame and composition. Large inner surface area can be obtained because of the inner void space of hollow structure. In recent years, hollow noble metals have been applied in the field of electrocatalyst due to the high electron transfer rate and large surface area [11]. However, few articles reported the applications of hollow noble metals in the field of biosensors.

In the present work, cubic PtCu NCs were fabricated using cuprous oxide (Cu_2_O) crystals as sacrificial templates, and their electrocatalytic activity towards H_2_O_2_ was investigated. The nonenzymatic H_2_O_2_ sensors exhibited excellent electrocatalytic performance with a high sensitivity and wide linear range for the determination of H_2_O_2_.

## Methods

### Reagents

Chloroplatinic acid, H_2_O_2_ (30 wt.% in H_2_O) and Nafion solution (5.0 wt.% in a mixture of lower aliphatic alcohols and water) were purchased from Sigma-Aldrich (St. Louis, MO, USA). All other reagents were of analytical grade and used as received without further purification (Chengdu Kelong, Chengdu, China). High-quality deionized water (resistivity > 18.0 MΩ cm^-1^) used for all experiments was prepared by a Water Purification System (UPT-II-10 T) provided by Chengdu YouPu, Chengdu, China.

### Preparation of PtCu NCs

Cubic Cu_2_O template was prepared according to the previous report [12]. Ten milliliters of NaOH aqueous solution (2 M) was added dropwise into the stirred CuCl_2_ · 2H_2_O (100 mL, 0.01 M) at 55°C. After stirring for 0.5 h, 10.0 mL ascorbic acid solution (0.6 M) was added. The final products were collected by centrifugation after 3 h, followed by drying in vacuum at 40°C for 24 h.

In order to prepare PtCu NCs, 10 mg prepared Cu_2_O was dispersed in 10 mL distilled water by ultrasonic for 15 min and then 40 mg sodium citrate was added. After stirring for 15 min, 1 mL chloroplatinic acid (20 mM) was added. After reacting for 20 min, 1 mL of dilute HNO_3_ (5 mM) was injected into the above solution to etch the Cu_2_O cores. After 40 min, the ultimate products were separated by mild centrifugation and dried at 40°C for 24 h in an oven.

### Electrochemical measurements

All electrochemical measurements were performed on a μIII Autolab electrochemical workstation in 0.1 M phosphate-buffered solution (PBS, pH = 7.0). Neutral PBS was obtained by mixing NaH_2_PO_4_ and Na_2_HPO_4_ solution (0.1 M). A conventional three-electrode system was used with Ag/AgCl (saturated with KCl) and platinum as the reference electrode and counter electrode, respectively. PtCu NC modified glassy carbon electrode (GCE, *Ф* = 3 mm) served as the working electrode. Typically, GCE was carefully polished with 0.05 μm alumina powders. Then, 5 μL of PtCu NC suspension (5 mg/mL) was cast onto the GCE and dried in air. Finally, 3 μL 1% Nafion solution was dipped onto the modified electrode.

## Results and discussion

### Characterizations

**Figure 1:**
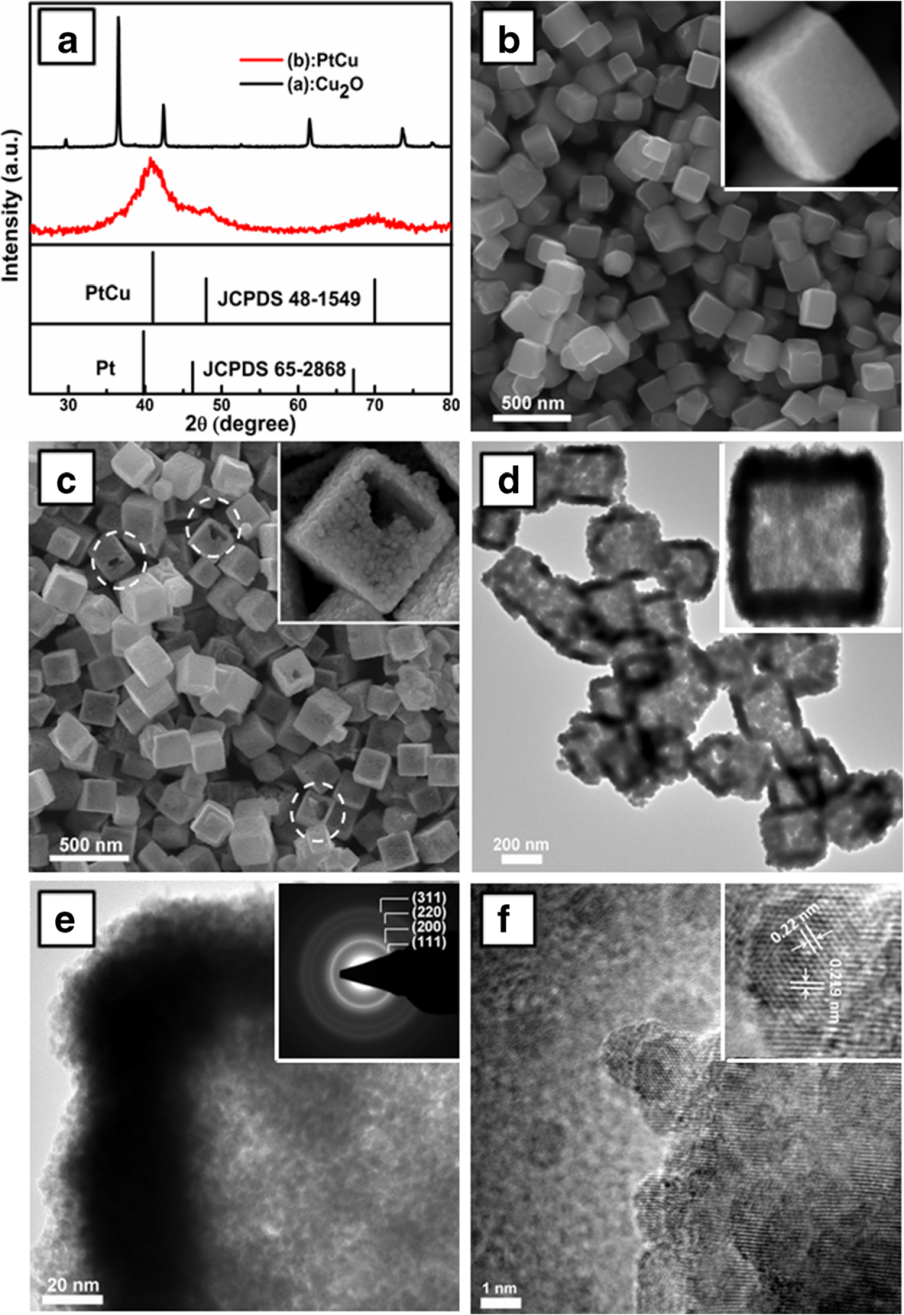
**XRD patterns and SEM, TEM, and HRTEM images.** XRD patterns of Cu_2_O and PtCu NCs **(a)**. SEM image of the Cu_2_O template **(b)** and PtCu NCs **(c)**. TEM **(d)** and HRTEM **(e, f)** images of the PtCu NCs. The insets of **(b)** and **(c)** are the SEM images of single Cu_2_O crystal and PtCu NC, respectively. The inset of **(d)** is the TEM image of a single PtCu NC. The insets of **(e)** and **(f)** are the SAED pattern and lattice fringes of PtCu NC, respectively.

As shown in Figure 1a, no Cu_2_O (JCPDS 65–3288) residue remains in the final products. Compared to pure Pt (JCPDS 65–2868), all diffraction peaks shift to large angle direction. The diffraction peaks located at around 41.2°, 48.1°, and 70° can be indexed to cubic PtCu alloy (JCPDS 48–1549). The average particle size of PtCu was calculated to be 2.9 nm according to the Scherrer equation:

(1) D=kλ/Bcosθ,

where *B* is the full width at half maximum (FWHM), *λ* is the X-ray wavelength (1.5406 Å), and *K* is a shape factor (about 0.89). On account of the fact that the Cu_2_O/Cu redox pair value is 0.36 V, which is much lower than that of PtCl_6_^2-^/Pt (0.735 V), therefore, Cu_2_O crystals can be used as the reducing agent and sacrificial template for the synthesis of cubic PtCu NCs. The formation process of PtCu NCs can be explained in the following equations:

(2) $$\begin{array}{l} 2{\mathrm{Cu}}_{2}O+4H^{+}+{{\mathrm{PtCl}}_{6}}^{2-}\to \mathrm{Pt}+4{\mathrm{Cu}}^{2+}+6{\mathrm{Cl}}^{-}\\ \;+2H_{2}O \end{array}$$

(3) $${\mathrm{Cu}}_{2}O+2H^{+}\to \mathrm{Cu}+{\mathrm{Cu}}^{2+}+H_{2}O$$

According to the above equations, the coexistence of Cu can be attributed to the disproportionation reaction of Cu (I). The reactions can simultaneously produce metallic Cu and Pt in the presence of H^+^, resulting in the formation of PtCu alloy. Figure 1b,c shows the scanning electron microscope (SEM) images of the prepared Cu_2_O template and the cubic PtCu NCs, respectively. The cubic Cu_2_O crystals have an average edge length about 200 nm, and the surface of the Cu_2_O crystals is smooth, uniform, and regular. However, the surface of PtCu NCs changes into rough and porous, indicating the formation of PtCu aggregates. It is clear that the PtCu NCs maintain the morphology of the Cu_2_O template and the interiors are hollow. The transmission electron microscope (TEM) image of PtCu NCs (Figure 1d) further provides convincing evidence of the hollow structure. For a further investigation, high-resolution transmission electron microscope (HRTEM) images were taken and displayed in Figure 1e,f. It is displayed that the shell of the PtCu NCs is porous (Figure 1e), and the porous shell is composed of amounts of PtCu nanograins. The porous structure possesses large specific surface area, which is beneficial for the electrocatalysis of H_2_O_2_. The inserted selected area electron diffraction (SAED) pattern indicates that the PtCu NCs have a polycrystalline structure. From Figure 1f, the size of the nanograins is about 2 to 4 nm, which agrees well with the value calculated from X-ray diffraction (XRD). The spacing for marked adjacent lattice fringes of PtCu NCs is about 0.22 nm, which is consistent with the standard value of PtCu (111) lattice spacing (0.219 nm).

### Electrochemical performances of the PtCu NC electrode

**Figure 2:**
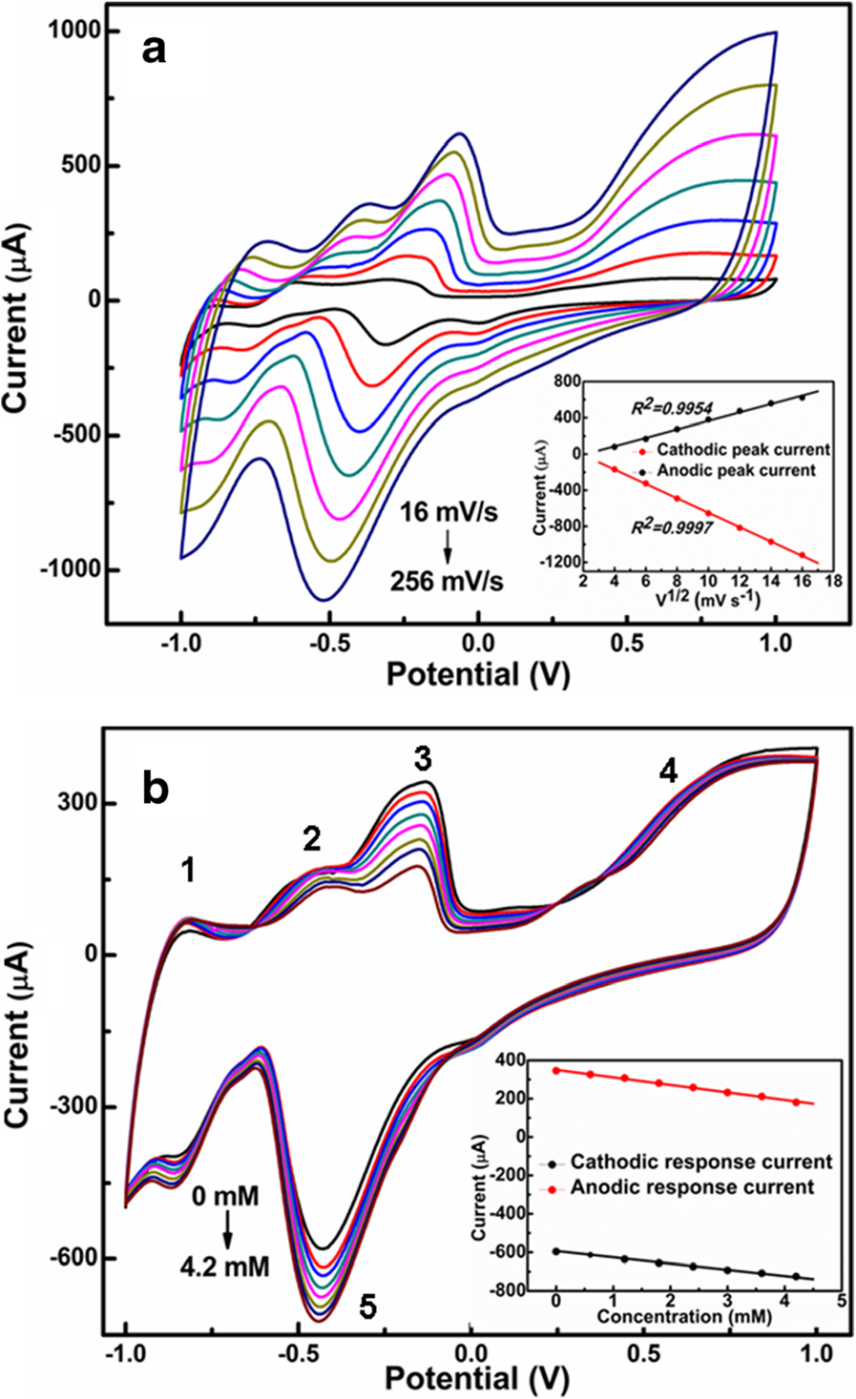
**CVs of PtCu NC electrode. (a)** CVs of PtCu NC electrode in 0.1 M PBS containing 1 mM H_2_O_2_ with different scan rates. The inset shows the relationship between the peak current and scan rate. **(b)** CVs of PtCu NC electrode in 0.1 M PBS with different concentrations of H_2_O_2_. The inset shows the dependence of the peak current on the concentration of H_2_O_2._

In order to estimate the kinetics of the electrode, the cyclic voltammetries (CVs) of cubic PtCu NC electrode were measured in 0.1 M PBS containing 1.0 mM H_2_O_2_ at different scan rates. As can be seen from Figure 2a, both the anodic and cathodic peak currents are proportional to the square root of the scan rate, indicating that the electrocatalytic process is diffusion-controlled. CVs of PtCu NC electrode in 0.1 M PBS with different concentrations of H_2_O_2_ were illustrated in Figure 2b. With the increase of the concentration, both the anodic and cathodic peak currents linearly change, showing a linear dependence between the peak current and the concentration of H_2_O_2._ As can be seen from Figure 2b, peaks 1 and 2 corresponding to hydrogen adsorption are clearly investigated. Peaks 3 and 4 are the oxidation peaks of Cu and Pt in the alloy, respectively. Peak 5 corresponds to metal oxide reduction. With the reduction of Pt, more active sites are obtained, and the response current is clearly investigated.

**Figure 3:**
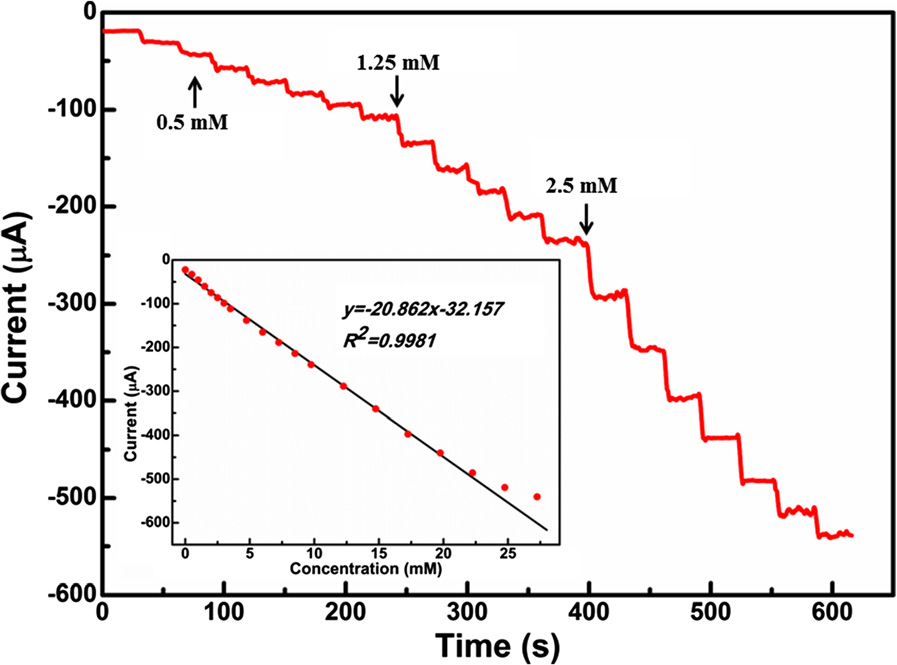
**Current-time response of PtCu NC electrode towards H**_**2**_**O**_**2**_**.** The inset shows the relationship between the catalytic current and the concentration of H_2_O_2_.

Figure 3 displays the amperometric response of the cubic PtCu NC electrode at -0.45 V to successive injection of a certain amount of H_2_O_2_ into the stirred 0.1 M PBS, and the corresponding calibration curve is exhibited in the inset. After the injection of H_2_O_2_ into the 0.1 M PBS, a well-defined, stable, and fast amperometric response was observed. The linear relationship was obtained for concentration ranging from 5 μM to 22.25 mM. The linear regression equation was given as *y =* -20.862*x* - 32.157 [*I* (μA); *x* (mM)], with a correlation coefficient of *R* = 0.9990. The detection limit of H_2_O_2_ was found to be 5 μM (S/N = 3) with a relatively high sensitivity of 295.3 μA mM^-1^ cm^-2^. The sensitivity and linear range obtained at PtCu NC electrode are better than those for HRP immobilized at multiwalled carbon nanotube/alumina-coated silica nanocomposite modified glassy carbon electrode (157 μA mM^-1^ cm^-2^; 1 to 500 μM) [13] and nano-Au monolayer supported by carbon ceramic electrode (290 μA mM^-1^ cm^-2^; 12.2 μM to 1.1 mM) [14]. The excellent performance may be attributed to the possible synergetic effect between Pt and Cu [15] and the porous structure of the PtCu NCs, which provide a large specific surface area. In terms of the synergetic effect, Cu atom in the PtCu alloy acts both as promoting centers for the generation of the Cu-OH_ad_ species and as an electron donor to Pt in the PtCu alloy. The incorporation of Cu atom decreases the Pt 4f binding energies and consequently reduces the Pt-OH_ad_ bond strength. Therefore, the intimate contact between Pt and Cu domains in the PtCu alloy greatly promotes the regeneration of Pt sites for high electrochemical activity towards hydrogen peroxide.

To estimate the effective surface area of the PtCu NC electrode, cyclic voltammograms on PtCu NC electrode in a solution containing 5 mM K_3_Fe(CN)_6_ and 0.1 M KCl were performed [16]. According to the Randles-Sevcik equation [17],

(4) $$I_{p}=2.69\times 10^{5}\times n^{3/2}AD^{1/2}v^{1/2}C,$$

where *A* is the effective surface area (cm^2^), *I*_p_ is the peak current of the redox reaction of [Fe(CN)_6_]^3-/4-^ (A), *n* is the number of electrons transferred (*n* = 1), *D* is the diffusion coefficient (0.76 × 10^-5^ cm^2^ s^-1^), *v* is the scan rate (V s^-1^), and *C* is the concentration of K_3_Fe(CN)_6_ (5 mM). The calculated value of *A* is 0.83 cm^2^ for the PtCu NC electrode, which is 11.75 times of the bare GCE.

## Conclusions

Cubic PtCu NCs were successfully synthesized using Cu_2_O as the template. The PtCu NC electrode exhibited excellent electrocatalytic activity towards H_2_O_2_. The observed detection limit and sensitivity for PtCu NC electrode was 5 μM and 295.3 μA mM^-1^ cm^-2^, respectively, with a wide linear range from 5 μM to 22.25 mM. On the basis of our research, the PtCu NC electrode has potential applications for the design of hydrogen peroxide sensor.

## Competing interests

The authors declare that they have no competing interests.

## Authors' contributions

LT designed the experiment and wrote the paper. XZ and WH prepared the solution and the modified electrode. BL carried out the synthesis of PtCu nanocage. YL did the electrochemical measurements. All authors read and approved the final manuscript.
